# Transmitted drug resistance and subtype patterns of viruses from reported new HIV diagnoses in Germany, 2017–2020

**DOI:** 10.1186/s12879-023-08649-3

**Published:** 2023-10-10

**Authors:** Uwe Fiebig, Britta Altmann, Andrea Hauser, Uwe Koppe, Kirsten Hanke, Barbara Gunsenheimer-Bartmeyer, Viviane Bremer, Axel Baumgarten, Norbert Bannert

**Affiliations:** 1 Unit 18 “HIV and other Retroviruses, Sexually transmitted bacterial Pathogens (STI) and HIV”, Department of Infectious Diseases, Robert Koch Institute, Berlin, Germany; 2 Unit 18 “HIV and other Retroviruses”, Department of Infectious Diseases, Robert Koch Institute, Berlin, Germany; 3 Unit 34 “HIV/AIDS, STI and Blood-borne Infections”, Department of Infectious Disease Epidemiology, Robert Koch Institute, Berlin, Germany; 4 Center for Infectiology Berlin–Prenzlauer Berg, Nordufer 20, 13353 Berlin, Germany

**Keywords:** HIV, Transmitted drug resistance, HIV subtype, Molecular surveillance, HIV diagnosis

## Abstract

**Background:**

The transmission of resistant HIV variants jeopardizes the effective use of antiretrovirals for therapy and prophylaxis. Molecular surveillance of new HIV diagnoses with a focus on prevalence and type of resistance associated mutations and the subtype of circulating viruses is mandatory.

**Method:**

From 2017 to 2020, 11,527 new HIV diagnoses were reported in Germany to the Robert Koch Institute (RKI). Protease (PR) and reverse-transcriptase (RT) sequences were obtained from 4559 (39.6%) cases, and PR, RT and integrase (IN) sequences were obtained from 3097 (26.9%) cases. The sequences were analyzed with data from the national HIV reports.

**Results:**

Among all cases in the analysis, the proportion of primary resistance was 4.3% for nucleoside reverse-transcriptase inhibitors (NRTIs), 9.2% for non-NRTI (NNRTIs), 3.3% for protease inhibitors (PIs) and 1.4% for integrase inhibitors (INIs). Dual-class resistance was highest for NRTIs/NNRTIs with 1.2%. There was no trend in the proportion of viruses resistant to drug classes. Most individual key mutations associated with relevant resistance had a prevalence below 1% including K65R (0.1%) and M184V (0.6%). A notable exception was K103NS, with a prevalence of 2.9% and a significant increase (p_Trend_=0.024) during 2017–2020. In this period, diagnoses of infections with HIV-1 subtype B were the most common at 58.7%, but its prevalence was declining (p_Trend_=0.049) while the frequency of minority subtypes (each < 1%) increased (p_Trend_=0.007). Subtype B was highest (75.6%) in men who have sex with men (MSM) and lowest in reported heterosexual transmissions (HETs, 22.6%).

**Conclusion:**

The percentage of primary resistance was high but at a stable level. A genotypic determination of resistance is therefore still required before the start of therapy. The subtype diversity of circulating HIV-1 is increasing.

## Introduction

The broader availability of combined antiretroviral therapy (cART) in the last decade has markedly reduced the incidence of HIV as well as the mortality and morbidity caused by the virus on a global scale. The goal of cART is a rapid and sustainable reduction of the viral load below the detection limit in an infected individual, which usually terminates the progression of the disease and permits recovery of the immune system. Moreover, effective virus suppression prevents sexual transmission [1]. According to recent surveillance data approximately 96% of the treated individuals in Germany are virally suppressed [2].

The basis of HIV surveillance in Germany is the reporting of new diagnoses anchored in the Protection Against Infection Act (Infektionsschutzgesetz, IfSG). This national epidemiological surveillance is supplemented by data obtained from the genome of the virus. The primary goals of this molecular scrutiny of newly diagnosed cases include the identification and analysis of transmitted resistance to approved antiretroviral therapeutics and subtyping of the circulating viral variants.

Transmitted drug resistance (TDR), also called primary resistance, is of high clinical relevance. The underlying resistance associated mutations restrict the portfolio of effective antiretroviral regimens. These mutations can lead to therapy failures or result in new infections in the context of preexposure or postexposure prophylaxis [3]. According to the Federal Joint Committee as well as national and international guidelines, genotypic resistance testing should be carried out before starting therapy [4, 5]. In Germany primary resistance testing includes nucleoside reverse transcriptase inhibitors (NRTIs), non-nucleoside reverse transcriptase inhibitors (NRTIs), protease inhibitors (PIs), and integrase inhibitors (INIs).

In addition to its clinical significance, monitoring primary HIV resistance is also relevant to public health. The extent of transmission of resistant viruses is a factor in defining and evaluating prevention strategies. It is also essential for keeping approved antiretrovirals effective or developing new or improved drugs to meet the public health goals of HIV/AIDS elimination. In this context, primary resistance to antivirals used in initial therapies and for preexposure prophylaxis (PrEP) plays an important role [6]. Both, the initial regimens and PrEP are only fully effective if the virus has no mutations that reduce the antiviral activity of the drugs. In Germany, it is predominantly men who have sex with men (MSM) that benefit from PrEP [7]. Data on the prevalence of primary resistance are also included in ECDC (European Centre for Disease Prevention and Control) and WHO (World Health Organization) surveys and in cost-benefit assessments of baseline resistance tests [6, 8–10].

The sequence information used for the genotypic determination of resistance can also be utilized for subtyping. Data on the virus subtype support the investigation of transmission chains and the geographic origin of an infection [11]. Molecular monitoring is typically restricted to determining the subtype of the HIV-1 group M virus [12, 13]. Infections with HIV-1 from other groups or with HIV-2 are very rare in Germany [11, 14]

In this study we examined the pattern and trends of TDR and HIV subtypes of the new diagnoses reported in 2017–2020. The analysis included a special focus on populations at risk of acquiring HIV. Our results update [12, 15] and extend [16] previously published findings [12, 15].

## Methods

### Sample material

Due to the national reporting obligation for relevant human pathogens and diseases specified in German law (IfSG), the RKI receives a notification of new HIV diagnoses. The IfSG enables supplementary projects for the molecular monitoring of circulating pathogens. In this context, a nationwide network of approximately 70 laboratories [17] reporting 50–60% of all new HIV diagnoses in the country submit plasma or serum to the RKI along with the report form for a case. Some laboratories send the sample material on a filter paper (Whatman 903) as dried serum or plasma spots. Before genotyping, RNA was extracted from these dried spots as previously described [15].

### Sample processing, genotypic determination of resistance and subtyping

Viral RNA was isolated from the sample material (serum, plasma or spot elution) using the automated Biomerieux EasyMag platform. The RNA was subsequently quantified and transcribed into cDNA [12]. The cDNA then served as a template for the amplification of three regions of the HIV genome, which contain all major resistance-associated positions. The genomic regions of protease (PR, amino acids 9–99), reverse transcriptase (RT, amino acids 1-252) and integrase (IN, amino acids 1-279) were amplified according to previously published protocols [12, 15]. The amplicons were sequenced using an Illumina NGS method (MiSeq). Resistance-associated mutations with a cutoff value of 20% were recorded [12, 15].

Resistance was determined and assessed using the Stanford HIV Drug Resistance Database version 8.9 algorithm [18, 19]. Sequences that achieved a mutation penalty score of over 10 (at least low-level resistance) were classified as resistant to a therapeutic agent [18].

The subtype of HIV-1 group M viruses was determined using the Stanford HIVdB, REGA HIV Subtyping and COMET HIV-1 tools [20, 21]. If a subtype or circulating recombinant form (CRF) could not be assigned unambiguously, a maximum-likelihood tree with bootstrap (IQ-TREE) was calculated using the HIV-1 subtype reference panel from the Los Alamos HIV sequence database [22]. As in our previous studies [12, 15], only subtype classifications with a bootstrap value of > 70% in the tree were considered. Otherwise the sequences were classified as unique recombinant forms (URFs).

For further analysis, the obtained molecular data were combined with sociodemographic information from the nameless notification form.

### Statistical analysis

Statistical analyses were performed using Excel (chi^2^ test, confidence intervals) and Stata, version 17.0 (Cochran–Armitage test for trend).

## Results

### Proportion of analyzed samples and characteristics of the study population

In the four-year period from 2017 to 2020, 11,527 new HIV diagnoses were reported to the RKI [14, 23]. PR and RT sequences were obtained from 4559 (39.6%) of these new diagnoses [16]. In 3097 (26.9%) of the reported cases, the relevant IN genome region could also be amplified and examined in addition (PR, RT, IN) [16]. The number and proportion of reported new diagnoses and the cases with PR/RT and PR/RT/IN sequences stratified by gender, transmission route and origin of the infected person are shown in Table 1.

**Table 1:** Characteristics of notified cases and the study population

Feature	Notification	PR/RT^a^	PR/RT/IN^a^
	N (%)	N (%)	N (%)
**Gender**			
Male	9016 (78.2)	3780 (82.9)	2575 (83.1)
Female	2492 (21.6)	771 (16.9)	516 (16.7)
Diverse/Not reported^b^	19 (0.2)	8 (0.2)	6 (0.2)
**Mode of transmission**			
MSM^c^	5465 (47.4)	2420 (53.1)	1659 (53.6)
HET^d^	2814 (24.4)	940 (20.6)	638 (20.6)
PWID^e^	583 (5.1)	214 (4.7)	146 (4.7)
Other	63 (0.5)	12 (0.3)	9 (0.3)
Not reported	2602 (22.6)	973 (21.3)	645 (20.8)
**Country of origin**			
Germany	6198 (53.8)	2649 (58.1)	1817 (58.7)
Other	4274 (37.1)	1499 (32.9)	1025 (33.1)
Not reported	1055 (9.2)	411 (9.0)	255 (8.2)

^a^Successfully amplified and analyzed genome regions.

^b^Because of low numbers these two groups were merged for reasons of data protection.

^c^Men who have sex with men.

^d^Persons with a heterosexual mode of transmission.

^e^Persons who inject drugs.

### Prevalence and trends of primary resistance among individuals newly diagnosed with HIV-1

Of the 4559 examined sequences of RT and PR genomic regions from newly diagnosed HIV cases, 194 (4.3% [95% CI, 3.7; 4.8]) demonstrated resistance to NRTIs, 420 (9.2% [95% CI, 8.4; 10.1]) were resistant to NNRTIs and 149 (3.3% [95% CI, 2.8; 3.8]) were resistant to PIs. The prevalence of INI resistance was significantly lower at 1.4% (42/3097 [95% CI, 0.9; 1.8]) (Table 2). In new diagnoses with HIV resistance to more than one drug class, NRTI/NNRTI dual resistance was the most common at 1.2% (53/4559 [95% CI, 0.9; 1.5]).

**Table 2:** Prevalence of transmitted drug resistance in the total sample and in subpopulations^a^

Drug class^b^	Total	Gender				Mode of transmission					Country of origin		
		Male	Female	Diverse/N. r.^c,d^		MSM^e^	HET^f^	PWID^g^	Other/N. r.^c,d^		Germany	Other	N. r.^d^
	N (%)	N (%)	N (%)	N (%)		N (%)	N (%)	N (%)	N (%)		N (%)	N (%)	N (%)
NRTI	194 (4.3)	159 (4.2)	34 (4.4)	1 (12.5)		103 (4.3)	43 (4.6)	6 (2.8)	42 (4.3)		109 (4.1)	73 (4.9)	12 (2.9)
NNRTI	420 (9.2)	327 (8.7)	92 (11.9)	1 (12.5)		198 (8.2)	111 (11.8)	19 (8.9)	92 (9.3)		220 (8.3)	162 (10.8)	38 (9.2)
PI	149 (3.3)	126 (3.3)	22 (2.9)	1 (12.5)		88 (3.6)	21 (2.2)	4 (1.9)	36 (3.6)		91 (3.4)	45 (3.0)	13 (3.2)
INI	42 (1.4)	39 (1.5)	3 (0.6)	0 (0.0)		22 (1.3)	12 (1.9)	0 (0.0)	8 (1.2)		25 (1.4)	16 (1.6)	1 (0.4)
NRTI + NNRTI	53 (1.2)	36 (1.0)	17 (2.2)	0 (0.0)		16 (0.7)	21 (2.2)	5 (2.3)	11 (1.1)		24 (0.9)	25 (1.7)	4 (< 0.1)
NRTI + PI	8 (0.2)	5 (0.1)	3 (0.4)	0 (0.0)		3 (0.1)	3 (0.3)	1 (0.5)	1 (0.1)		3 (0.1)	5 (0.3)	0 (0.0)
NRTI + INI	3 (< 0.1)	3 (0.1)	0 (0.0)	0 (0.0)		2 (0.1)	0 (0.0)	0 (0.0)	1 (0.2)		2 (0.1)	1 (< 0.1)	0 (0.0)
NNRTI + PI	26 (0.6)	20 (0.5)	5 (0.6)	1 (12.5)		17 (0.7)	5 (0.5)	1 (0.5)	3 (0.3)		16 (0.6)	10 (0.7)	0 (0.0)
NNRTI + INI	2 (< 0.1)	2 (< 0.1)	0 (0.0)	0 (0.0)		0 (0.0)	0 (0.0)	0 (0.0)	2 (0.3)		0 (0.0)	1 (< 0.1)	1 (0.4)
PI + INI	1 (< 0.1)	1 (< 0.1)	0 (0.0)	0 (0.0)		1 (< 0.1)	0 (0.0)	0 (0.0)	0 (0.0)		0 (0.0)	1 (< 0.1)	0 (0.0)
NRTI + NNRTI + PI	4 (< 0.1)	3 (< 0.1)	1 (0.1)	0 (0.0)		2 (< 0.1)	1 (0.1)	1 (0.5)	0 (0.0)		2 (< 0.1)	2 (0.1)	0 (0.0)

^a^The number of analyzed cases belonging to a subpopulation is given in Table 1.

^b^Drug classes with INI are based on 3097 analyzed cases, all others are based on 4559 cases.

^c^Because of low numbers these two groups were merged for reasons of data protection.

^d^N.r.=Not reported.

^e^Men who have sex with men.

^f^Persons with a heterosexual mode of transmission.

^g^Persons who inject drugs.

Among the 3097 cases analyzed that had sequence data for PR/RT/IN, the proportion of resistant HIV was 15.2% as previously published [16]. Resistance frequency was similar between males, at 15.1% (388/2575), and females at 15.7% (81/516) (p = 0.716). HIV resistance to NNRTIs was significantly (p = 0.004) higher in women than in men, at 11.9% (92/771) versus 8.7% (327/3780) (Table 2).

Stratified by the mode of transmission, we observed a lower prevalence of HIV resistance in persons who inject drugs (PWID) (9.6%, 14/146) compared to those with heterosexual transmission (HET) (16.3%, 104/638, p = 0.041). Compared to HET, HIV resistance to NNRTIs was less common in MSM (8.2% vs. 11.8%, p = 0.001), and PI resistance was more common (3.6% vs. 2.2%, p = 0.039) (Table 2). Moreover, the overall primary HIV resistance was not significantly different in persons of German and non-German origin, with 14.8% (268/1817) and 16.3% (167/1025) (p = 0.273), respectively. However, resistance to NRTIs and NNRTIs was less commonly observed among people of German origin (4.1% [109/2649] versus 4.9% [73/1499], p < 0.001 and 8.3% [220/2649] versus 10.8% [162/1499], p = 0.007) (Table 2).

**Fig. 1:**
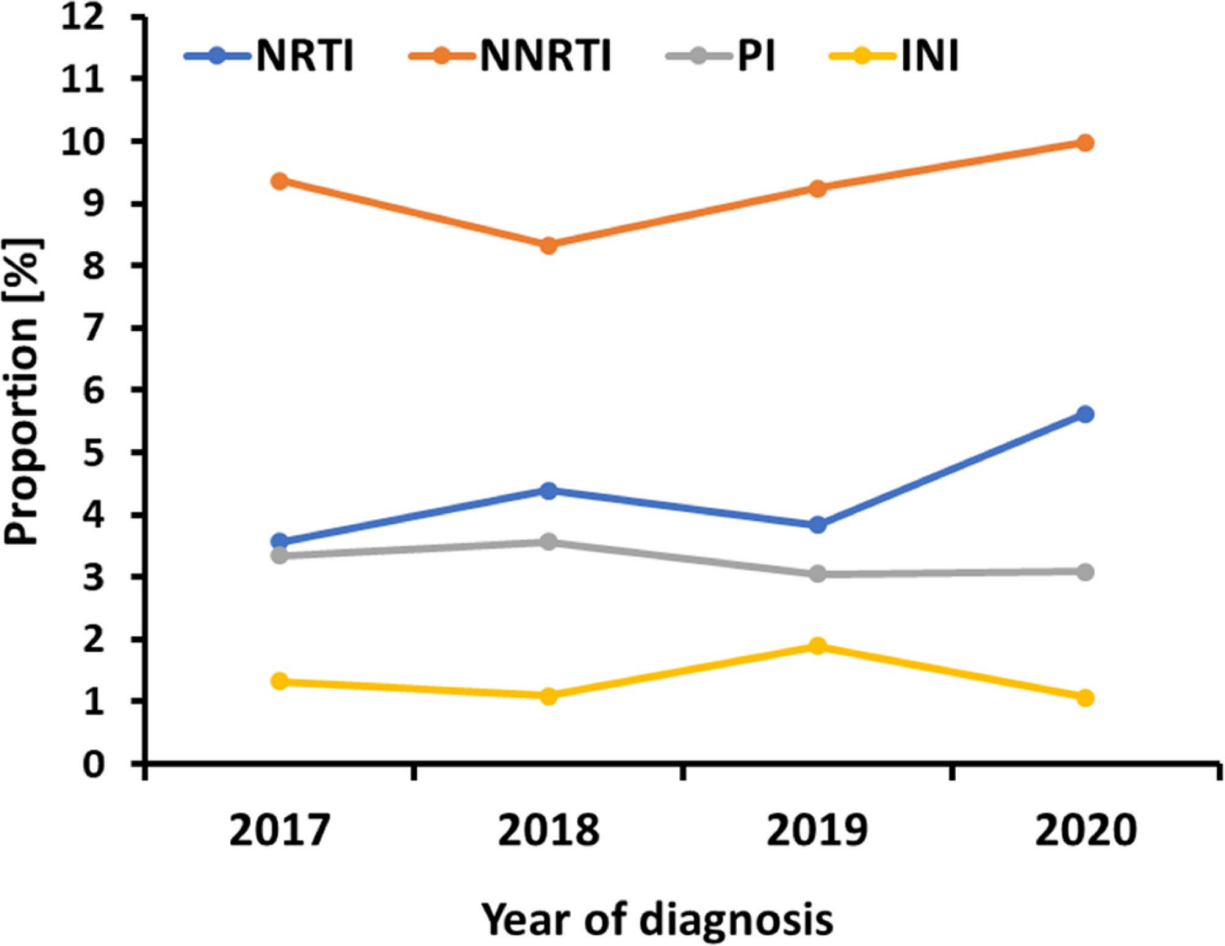
Proportion of primary resistance in the individual drug classes over time. N = 4559 for NRTI, NNRTI and PI; N = 3097 for INI

The prevalence of primary resistance to the four drug classes analyzed remained largely constant from 2017 to 2020 (Fig. 1).

The most common resistance-associated mutations were E138A at 3.6% (166/4559), resulting in low-level resistance to the NNRTI rilpivirine (RPV) and K103NS at 2.9% (133/4559) conferring resistance to NNRTI first-generation drugs such as efavirenz (EFV) and nevirapine (NVP) (Table 3).

**Table 3:** Prevalence of relevant and frequent resistance-associated mutations

NRTI		NNRTI		PI		INI	
Mutation	N (%)^a^	Mutation	N (%)^a^	Mutation	N (%)^a^	Mutation	N (%)^b^
M41L	51 (1.1)	A98G	11 (0.2)	V11I	58 (1.3)	T66I	2 (< 0.1)
K65R	5 (0.1)	K101EP	26 (0.6)	D30N	2 (< 0.1)	E92GQ	1 (< 0.1)
D67EGN	18 (0.4)	K103NS	133 (2.9)	M46IL	42 (0.9)	E138K	4 (< 0.1)
T69D	7 (0.2)	V108I	26 (0.6)	I50LV	0 (0.0)	G140AS	1 (< 0.1)
K70E	7 (0.2)	E138A	166 (3.6)	F53LY	11 (0.2)	Y143CHR	1 (< 0.1)
V75AMST	3 (< 0.1)	Y181CIV	21 (0.5)	Q58E	37 (0.8)	S147G	1 (< 0.1)
Y115F	2 (< 0.1)	Y188CHL	10 (0.2)	T74S	87 (1.9)	Q148HR	0 (0.0)
M184V	27 (0.6)	G190AES	33 (0.7)	V82ACFLTMS	23 (0.5)	N155HS	0 (0.0)
T215CDEFISVY	97 (2.1)	H221Y	8 (0.2)	I85V	4 (< 0.1)	S230R	0 (0.0)
K219ENQR	24 (0.5)	P225H	9 (0.2)	L90M	21 (0.5)	R263K	1 (< 0.1)

^a^NRTI, NNRTI and PI are based on 4559 analyzed cases.

^b^INI is based on 3097 analyzed cases.

**Fig. 2:**
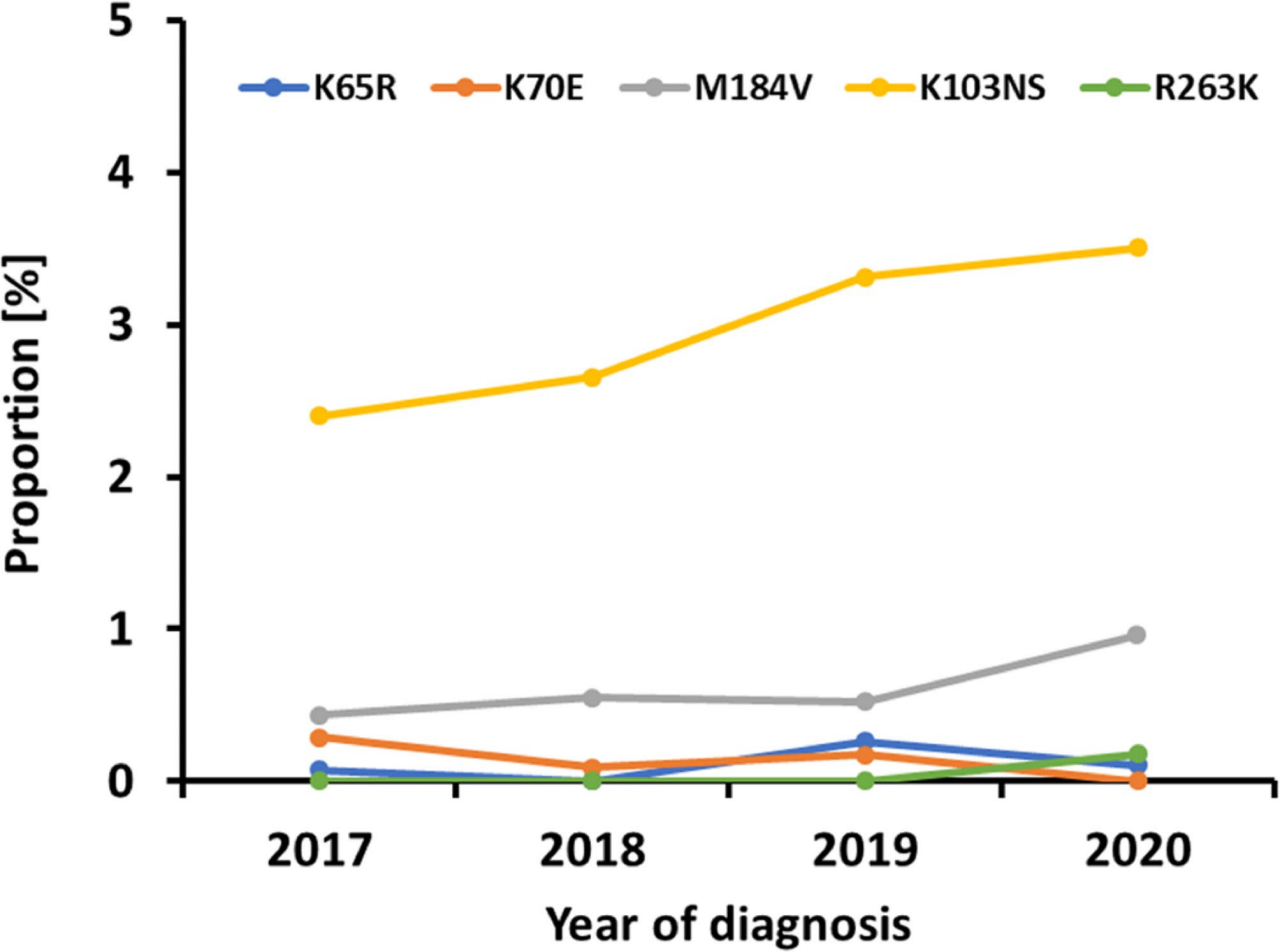
Prevalence of five key mutations over the analysis period. Key mutations include K103NS because of a significant increase and those that substantially decrease susceptibility to drugs recommended for first-line therapy in Germany [4]. N = 4559 for K65R (NRTI), K70E (NRTI), M184V (NRTI) and K103NS (NNRTI), N = 3097 for R263K (INI).

K103NS mutations significantly increased from 2.4% (33/1377) to 3.5% (33/942) (p_Trend_=0.024) (Fig. 2) in 2017–2020. T215 revertant substitutions (T215CDEFISVY), which are considered thymidine analog mutations (TAMs) and cause resistance to older NRTIs such as zidovudine (AZT) or stavudine (d4T), were also very common at 2.1% (97/4559) (Table 3). Among the key mutations with high relevance for current initial therapies, the non-TAM NRTI substitution M184V was the most common at 0.6% (27/4559) and reached a proportion of 1% (9/942) in 2020 (Table 3; Fig. 2). In particular, this mutation causes high-level resistance to emtricitabine (FTC) and lamivudine (3TC). FTC is approved for PrEP in a combination with tenofovir disoproxil fumarate (TDF) or tenofovir disoproxil alafenamide (TAF). K65R mutations affecting TDF and TAF were at a stable low level of 0.3% and below in 2017–2020 (Fig. 2).

### Prevalence of HIV-1 subtypes

**Fig. 3:**
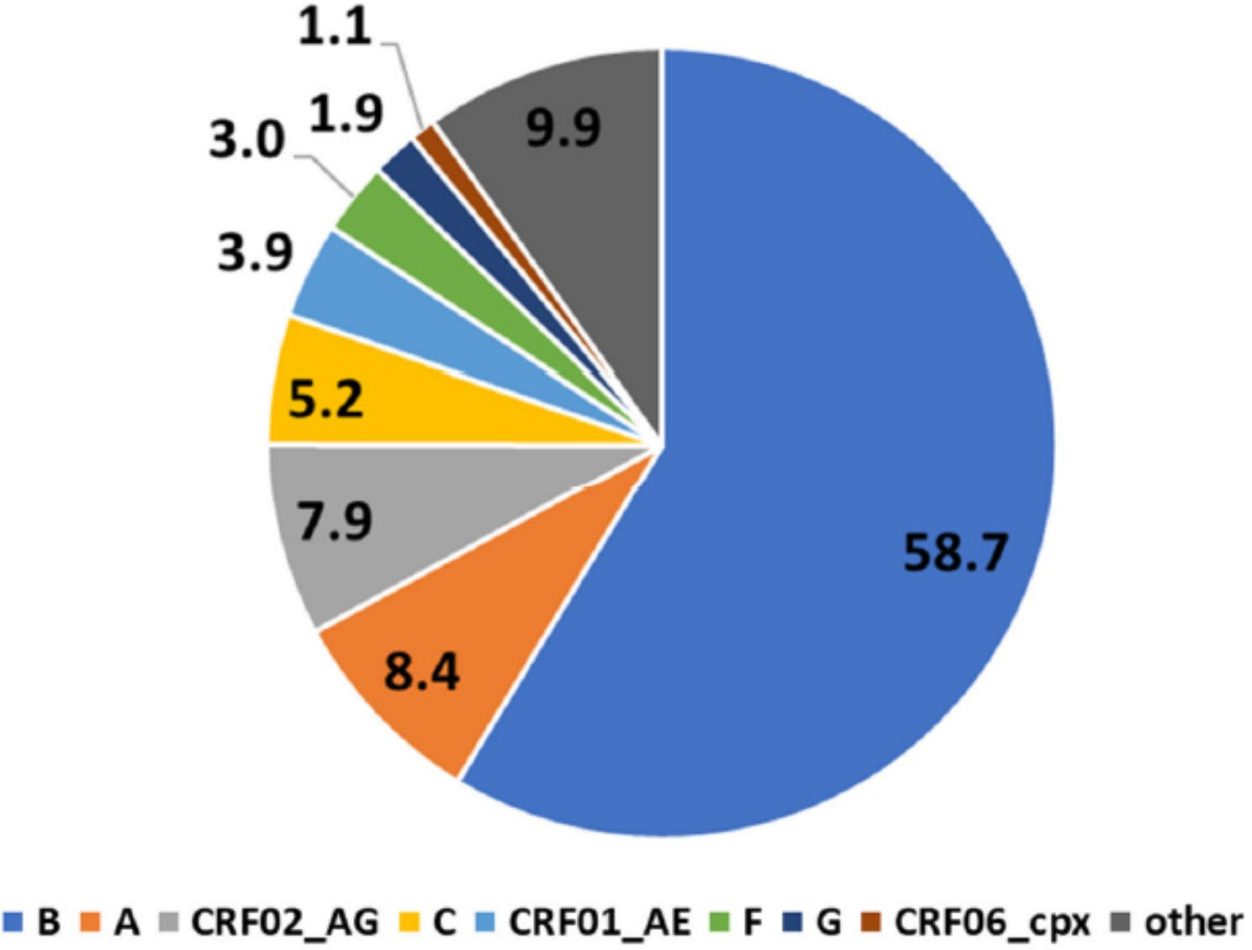
Prevalence of HIV-1 subtypes among new HIV diagnoses from 2017–2020. N = 4559; CRF, circulating recombinant form; cpx, complex

By far, the most common subtype among the 4559 new HIV-1 diagnoses analyzed was subtype B with 58.7%, followed by subtype A (8.4%), the recombinant form CRF02_AG (7.9%) and subtype C (5.2%). Approximately 10% of the examined HIV-1 infections were caused by subtypes and recombinant forms of which the individual mean prevalence was below 1% (Fig. 3).

**Fig. 4:**
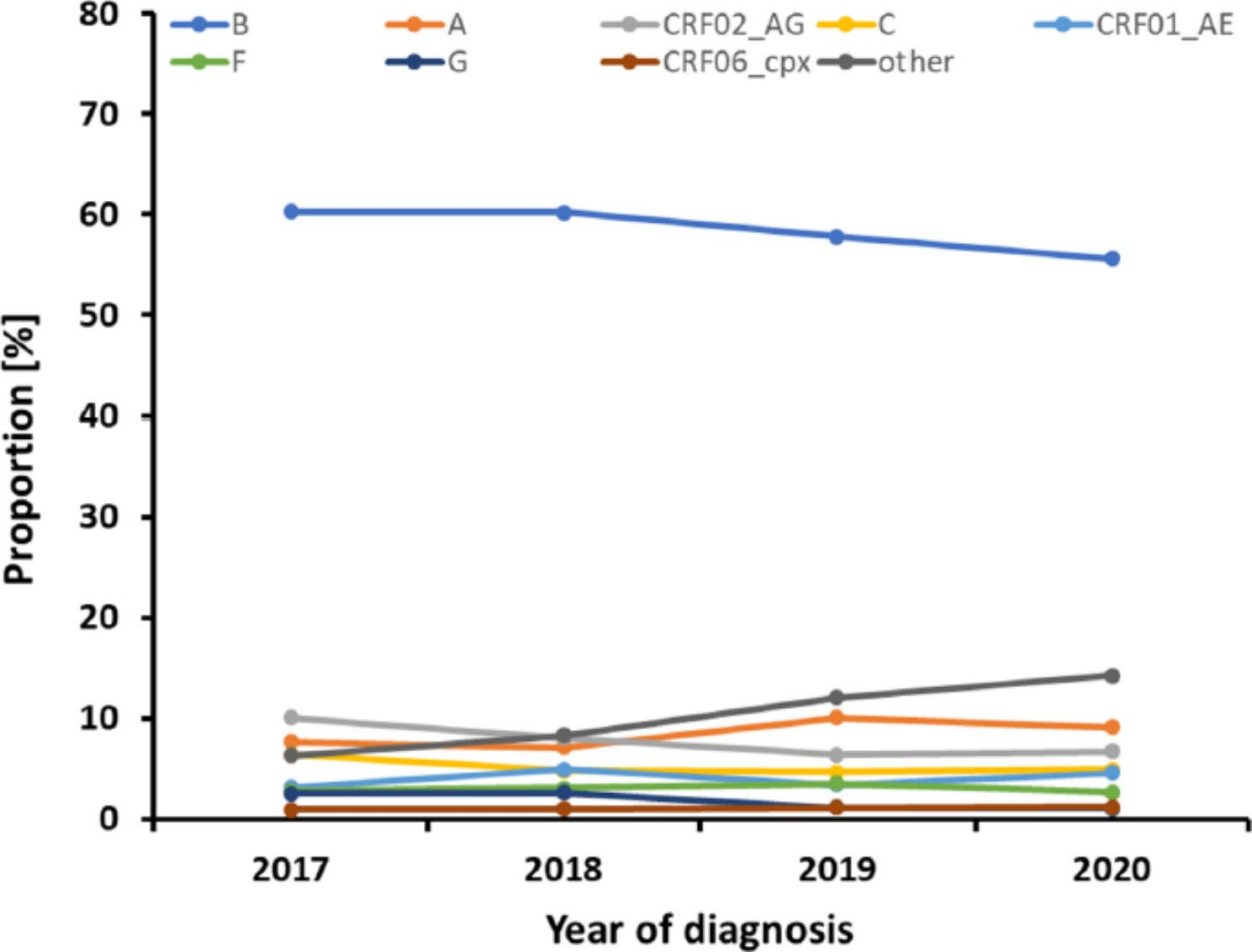
Prevalence of relevant (> 1%) subtypes over the analysis period. N = 4559; CRF, circulating recombinant form

While the proportion of subtype B diagnoses fell continuously between 2017 and 2020 from 60.3 to 55.6% (p_Trend_=0.049), the prevalence of subtype variants with frequencies < 1% increased from 6.3 to 14.2% (p_Trend_=0.007) (Fig. 4).

**Fig. 5:**
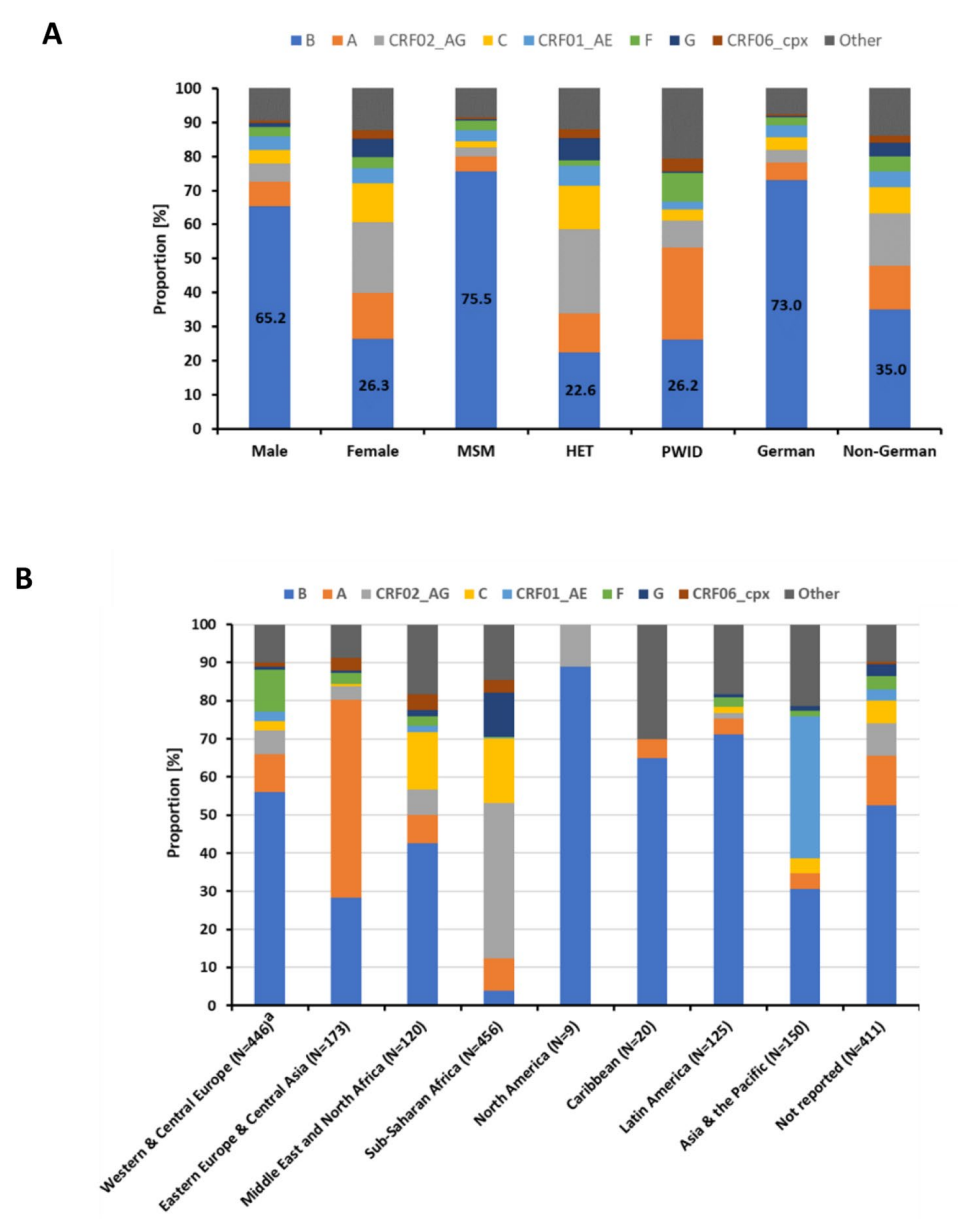
Prevalence of subtypes in subgroups. (**A**) Percentage of subtypes stratified by gender, transmission group and German or non-German origin. (**B**) Percentage of subtypes in cases with non-German origin stratified by region. CRF, circulating recombinant form; cpx, complex; MSM, men who have sex with men; HET, persons with a heterosexual mode of transmission; PWID, persons who inject drugs; ^a^without Germany

Subtype B was highly prevalent in MSM (75.6%) and in newly diagnosed cases of German origin (73.0%) (Fig. 5A). Subtype A, the second most frequent subtype, was common in PWID (27.1%) and CRF02_AG was common in HETs (24.7%) (Fig. 5A). Subtype A dominated cases with origins in Eastern Europe and Central Asia (52.0%) while CRF02_AG was very frequent in cases with origins in Sub-Saharan Africa (40.8%) (Fig. 5B). Low-prevalence subtypes reached high proportions in cases with origins in the Caribbean, Asia and the Pacific, and the Middle East and North Africa (Fig. 5B).

## Discussion

Ensuring effective HIV therapy and a reliable prevention of transmission using PrEP is a central public health interest. [8, 24–27]Therefore, HIV drug resistance is monitored at the national level in many countries. A molecular surveillance of new HIV diagnoses was established at the RKI in 2013 to monitor the problem of resistance and to analyze the virus variants circulating in Germany [12]. Primary resistance with potential clinical implications was diagnosed in 15.2% of the analyzed new cases from 2017 to 2020, as recently reported [16]. A similarly high prevalence of 14.5% was found in a pilot study for implementing a Europe-wide surveillance system with new diagnoses from 2015 in samples from nine countries [6]. In a comparable study between 2014 and 2018 in the USA, 18.9% of new HIV diagnoses showed resistance mutations [28].

Primary resistance to more than one drug class was rare in our survey, with the exception of NRTI/NNRTI dual resistance (1.2%). It was significantly higher in individuals of non-German origin (1.7%) in contrast to people of German origin 0.9% (p < 0.001). Of the 25 non-German cases with NRTI/NNRTI dual resistance, 11 were of African origin, where NNRTI use is much more common in many countries than in Germany [25, 29].

Approximately 40% of cases with primary resistance showed reduced susceptibility to older drugs that are rarely used in Germany and that are no longer recommended for initial therapy [4, 16, 30]. This resistance is primarily based on the TAMs and the polymorphic E138A variant in NRTI resistance and the K103NS mutation in NNRTI resistance. The proportion of these mutations is very consistent over long periods of time. This persistence can be largely explained by the level of viral fitness deficits. Especially for TAM pattern 2 and K103N mutations, the deficit is very low in comparison to the wild-type virus. [31, 32]. The prevalence of resistance due to substitutions at the K103 position is steadily increasing, a trend that has been observed and analyzed since 2014 in Germany [15]. Among drugs used in initial regimens, mutations that lead to pronounced resistance to the frequently prescribed non-TAM NRTIs (especially TDF/TAF, FTC, 3TC, ABC), as well as to PIs and INIs, are much more significant [30]. The frequency of these mutations is much lower and largely stable, ensuring sustained efficiency of the currently recommended primary regimens in the near future. This will be further supported by the approval of new therapeutics.

The development of primary resistance to TDF/TAF and FTC is particularly noteworthy. These active ingredients are part of many initial therapies and are approved for PrEP in Germany. TDF resistance is often caused by the K65R mutation, and FTC resistance is caused by the M184V mutation. Previously, we reported a prevalence of below 0.5% for the K65R and 0.5% for the M184V mutation for 2013–2016 [15]. The prevalence of these mutations in 2017–2020 was again below 0.5% for K65R and 0.6% for M184V showing no significant increase or decrease.

The analysis of the circulating subtypes revealed that the subtype B prevalence continued to decrease to 58.7% on average in the analysis period. In the four previous years, the proportion was significantly higher at 68.6% [15], and in 2013–2014, it was 77%. In contrast, the proportion of subtypes and recombinant forms with a prevalence of < 1% is increasing sharply. Also affected by this trend are new HIV infections among MSM. As a transmission group, MSM still have the highest subtype B prevalence, which is reminiscent for the initial spread in the 1980s. The decrease in the proportion of subtype B infections can, at least in part, be explained by the decrease of new diagnoses in the MSM transmission group and the increase of the PWID and HET groups in 2017–2020 in Germany [14]. The declining proportion among MSM is associated with an increase in PrEP usage [7]. As in other countries around the world, the HIV-1 epidemic is becoming more diverse in Germany [12, 15, 33, 34].

The study is not without limitations. (i) Data reported on the notification form are based on information originally received from the patient. Patients can give inaccurate information, particularly regarding the mode of transmission or previous HIV diagnoses, to the doctor. (ii) Although, measures for the exclusion of falsely reported new infections are in place (IfSG § 10(4)) we cannot completely rule out that cases of acquired resistance that have already been diagnosed and treated abroad may have been misinterpreted as primary resistance if the viral load allows amplification. (iii) Sample material from diagnoses that contain no or very low viral loads leads to deviations in the proportion of reported and molecularly analyzed cases. These cases are included in the report, but most were not included in the molecular analysis. Differences in this regard become particularly clear in the stratification according to characteristics (origin, sex, transmission path) (Table 1). Other reasons for differences cannot be ruled out. Due to these limitations, molecular monitoring is not representative of the new HIV diagnoses made in Germany and reported to the RKI. (iv) The proportions of successfully amplified integrase regions (largest amplicon) and analyzed cases for INI resistance are smaller than those for NRTIs, NNRTIs and PIs, and the statements are therefore less robust. (v) In contrast to the SDRM list utilized in other studies on transmitted resistance mutations, the Stanford HIVdb algorithm we applied is regularly updated but includes polymorphisms.

In conclusion, the proportion of new HIV diagnoses carrying resistance associated mutations to the main drug classes remained largely stable from 2017 to 2020. The result supports the current recommendations and guidelines for genotypic resistance testing, therapy and PrEP. The prevalence of subtype B viruses circulating in Germany continues to decrease, and subtype diversity increases. National population-level monitoring of circulating HIV remains critical for informing future recommendations for treatment and prevention as well as for strategies to end HIV/AIDS.

## Data Availability

All datasets including sequences are available from the corresponding author upon reasonable request.
